# The Evolution and Global Spatiotemporal Dynamics of Senecavirus A

**DOI:** 10.1128/spectrum.02090-22

**Published:** 2022-10-31

**Authors:** Huiguang Wu, Chen Li, Yongchen Ji, Chunxiao Mou, Zhenhai Chen, Jingwen Zhao

**Affiliations:** a College of Veterinary Medicine, Yangzhou University, Yangzhou, Jiangsu Province, China; b Joint International Research Laboratory of Agriculture and Agri-Product Safety, the Ministry of Education of China, Yangzhou University, Yangzhou, Jiangsu Province, China; c Jiangsu Co-Innovation Center for Prevention and Control of Important Animal Infectious Diseases and Zoonoses, Yangzhou University, Yangzhou, Jiangsu Province, China; d College of Animal Science and Technology, Yangzhou University, Yangzhou, Jiangsu Province, China; Changchun Veterinary Research Institute

**Keywords:** senecavirus A, evolution, phylogeny, selection, spatiotemporal dynamics

## Abstract

Recurrent outbreaks of senecavirus A (SVA)-associated vesicular disease have led to a large number of infected pigs being culled and has caused considerable economic losses to the swine industry. Although SVA was discovered 2 decades ago, knowledge about the evolutionary and transmission histories of SVA remains unclear. Herein, we performed an integrated analysis of the recombination, phylogeny, selection, and spatiotemporal dynamics of SVA. Phylogenetic analysis demonstrated that SVA diverged into two main branches, clade I (pre-2007 strains) and clade II (post-2007 strains). Importantly, analysis of selective strength showed that clade II was evolving under relaxed selection compared with clade I. Positive selection analysis identified 27 positive selective sites, most of which are located on the outer surface of capsid protomer or on the important functional domains of nonstructure proteins. Bayesian phylodynamics suggested that the estimated time to the most recent common ancestor of SVA was around 1986, and the estimated substitution rate of SVA was 3.3522 × 10^−3^ nucleotide substitutions/site/year. Demographic history analysis revealed that the effective population size of SVA has experienced a gradually increasing trend with slight fluctuation until 2017 followed by a sharp decline. Notably, Bayesian phylogeographic analysis inferred that Brazil might be the source of SVA’s global transmission since 2015. In summary, these data illustrated that the ongoing evolution of SVA drove the lineage-specific innovation and potentially phenotypically important variation. Our study sheds new light on the fundamental understanding of SVA evolution and spread history.

**IMPORTANCE** Recurrent outbreaks and global epidemics of senecavirus A-associated vesicular disease have caused heavy economic losses and have threatened the development of the pig industry. However, the question of where the virus comes from has been one of the biggest puzzles due to the stealthy nature of the virus. Consequently, tracing the source, evolution, and transmission pattern of SVA is a very challenging task. Based on the most comprehensive analysis, we revealed the origin time, rapid evolution, epidemic dynamics, and selection of SVA. We observed two main genetic branches, clade I (pre-2007 strains) and clade II (post-2007 strains), and described the epidemiological patterns of SVA in different countries. We also first identified Brazil as the source of SVA’s global transmission since 2015. Findings in this study provide important implications for the control and prevention of the virus.

## INTRODUCTION

Senecavirus A (SVA), known as Seneca Valley virus, belongs to the genus *Senecavirus* in the family *Picornaviridae*. SVA was initially discovered incidentally in 2002 as a contaminant in cell culture medium during cultivation of PER.C6 (embryonic retinal) cells (1). SVA has been detected in wild boar, mice, houseflies, and buffalo (2, 3). In addition to direct contact, fecal-oral transmission might be an important route of SVA transmission (4). In addition, on-farm employee entry, transportation, feed, parturition, and semen were identified as high-risk factors for SVA transmission (5, 6).

Although sequence analysis of archived picorna-like viruses revealed that circulation of SVA had begun in the swine population of the United States from at least 1988 (7, 8), SVA-caused vesicular disease was not detected in swine herds until 2007 (9), and only sporadic cases of SVA infection were reported during more than a decade since its discovery. At the end of 2014 and the beginning of 2015, a sudden large-scale outbreak of SVA associated with vesicular disease in pigs was first reported in Brazil (10, 11) and was then successively reported in the United States (12), Canada (13), Colombia (14), China (15), Thailand (16), and Vietnam (17). It is noteworthy that SVA infection causes frequent outbreaks of vesicular disease in pig herds in major swine-producing countries around the world. There have been three waves of SVA infection in pig herds in southern Brazil (18). Due to the severe economic losses caused by SVA-associated vesicular disease, the impact of this virus upon swine production is a worldwide concern.

The clinical features of SVA infection are clinically indistinguishable from those of other vesicular diseases, e.g., foot-and-mouth disease (FMD), swine vesicular disease (SVD), vesicular stomatitis (VS), and vesicular exanthema of swine (VES) (19). SVA can cause lameness, vesicles, and erosions on the coronary bands and snout (7). Additionally, the clinical manifestations in SVA-infected pigs at different growth stages are different. In neonatal piglets, SVA infection led to sudden death, cutaneous hyperemia, acute diarrhea, excessive salivation, lethargy, and neurological signs (20). The drowsiness, lameness, oral blisters, diarrhea, and hoof lesions were observed in SVA-infected weaned piglets (21). Finishing pigs infected by SVA presented lethargy, lameness, and vesicular lesions on the snout and feet (4).

SVA is a nonenveloped, icosahedral virus with a positive-sense, single-stranded, nonsegmented linear RNA genome. The genome is about 7.3 kb in length and includes a single open reading frame (ORF) flanked by 5′ and 3′ untranslated regions (UTRs). The 5′ UTR contains a type IV internal ribosome entry site (IRES) that initiates cap-independent translation without any host initiation factors (1). The ORF encodes a polyprotein composed of 2,181 amino acids, which is cleaved by viral protease into four structural proteins (VP1 to VP4) and eight nonstructural proteins (L, 2A, 2B, 2C, 3A, 3B, 3C, and RNA-directed RNA polymerase [RdRp]) (1). As a member of the family *Picornaviridae*, SVA is thought to have structural proteins with similar biological functions to those of other picornaviruses.

Although SVA has been studied for many years, its evolutionary characteristics and transmission history have not yet been fully investigated. Some studies reported that SVA might be divided into one (22), two (3, 23–25), or three (26, 27) main clades, but no definitive conclusion has been drawn. Given the potential threat of SVA to the global pig industry, tracking sources and transmission pathways of SVA-associated vesicular disease is crucial for epidemic prevention and control. Here, we performed a comprehensive analysis, including recombination detection and phylogenetic, phylodynamic, phylogeographic, and selection analyses, to illustrate the origin, evolution, and spatiotemporal pattern of SVA.

## RESULTS

### Recombination analysis.

A total of 249 SVA complete genomes were obtained from GenBank in December 2021 (see Table S1 in the supplemental material). The data set includes 14 sequences of SVA strains isolated from our laboratory. Eleven SVA strains with identical coding sequences (CDSs) were excluded from further analysis. Recombination analysis showed that five SVA strains (GD04/2017, SVA/CHN/10/2017, HeNNY-1/2018, HeNKF-1/2018, and CH-GDMZ-2019) were identified as recombinants (see Table S2 in the supplemental material). Each of the recombinants encompassed two or three isolates, which were suggested as the representatives of its putative parental lineages. Similarity plots produced the same results as those obtained from recombination detection program 4 (RDP4) (see Fig. S1 in the supplemental material). Interestingly, SVA recombinants identified in this study were isolated from China, and no recombinants were detected in strains from the other countries. After removing the recombinant genomes, a total of 233 SVA strain sequences were retained for further analysis, including those isolated from the United States (*n* = 103; 44.21%), China (*n* = 99; 42.49%), Canada (*n* = 14; 6.01%), Brazil (*n* = 10; 4.29%), Thailand (*n* = 5; 2.15%), Colombia (*n* = 1; 0.43%), and Vietnam (*n* = 1; 0.43%).

### Two highly divergent clades of SVA.

**FIG 1:**
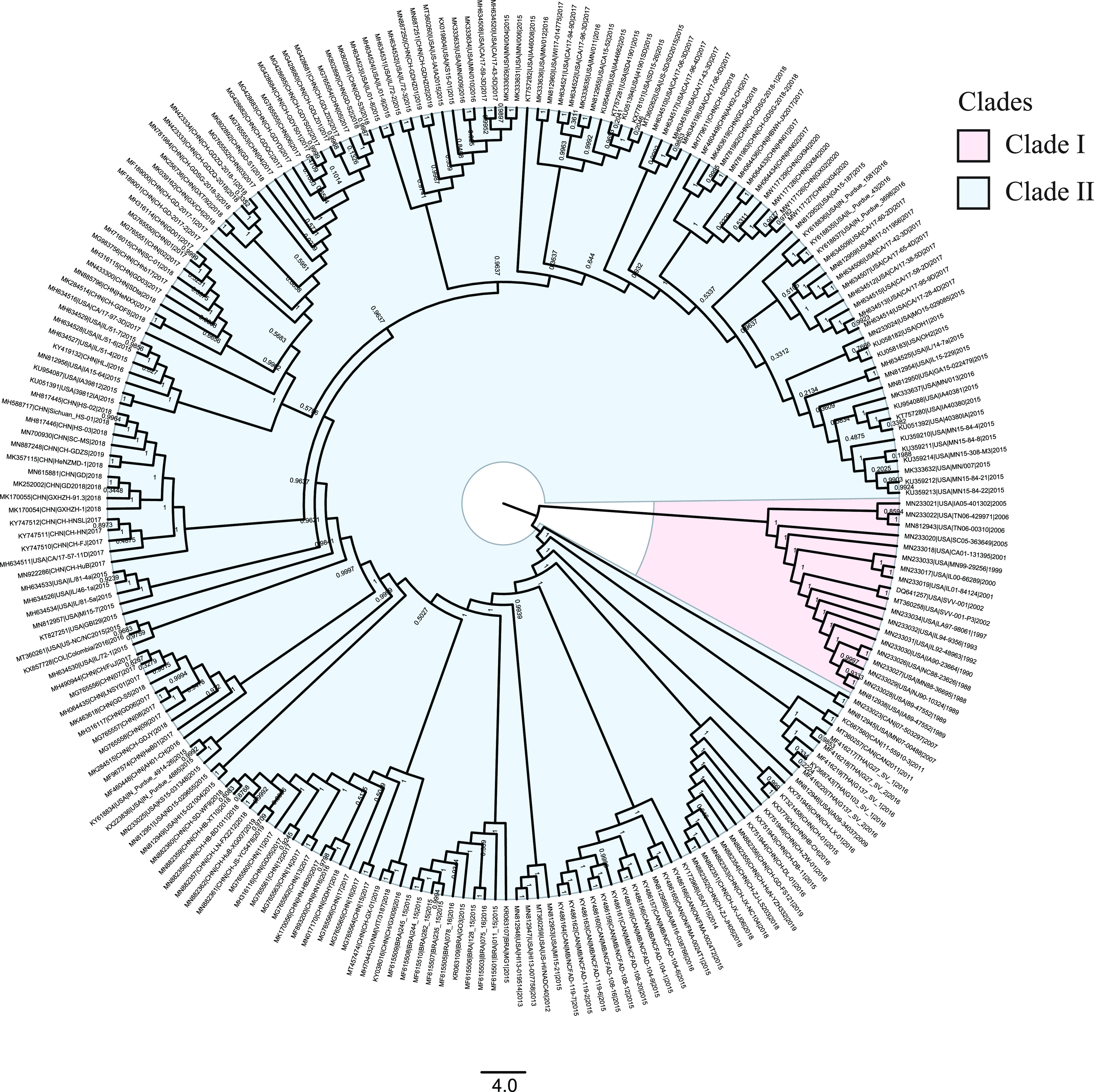
Bayesian inference tree of SVA genomes. The red block represents clade I isolated pre-2007. The blue block represents clade II isolated post-2007. The tree is rooted at its midpoint. Bayesian posterior probability values are shown at each node. Scale bar indicates average number of nucleotide substitutions per site.

Likelihood mapping analysis showed that the SVA data set displayed 92.4% fully resolved phylogeny (see Fig. S2 in the supplemental material), confirming that the data set was suitable for further analysis. The nucleotide saturation test (Xia's test) showed little saturation in the SVA data set (observed index of saturation substitution [*I*_ss_] < critical index of substitution saturation [*I*_ss.c_]; *P *< 0.05), indicating the suitability of this data set for further phylogenetic analysis. The Bayesian inference (BI) phylogeny of SVA genomes revealed two distinct and well-supported (high posterior probabilities ≥ 0.99) clades, here defined as clades I and II (Fig. 1). Clade I was represented by 19 strains collected from the United States from 1988 to 2006, while clade II was comprised of the isolates collected from seven countries since 2007. The genetic distance between clades I and II was 0.0713 ± 0.0030. The result of the two-sample *t* test showed that the genetic sequence diversities between clades I and II were significantly different (*Z* test *P = *0.00188; *T* test *P = *0.009514), implying significant genetic difference between two clades.

PhyloPart analysis of SVA identified the existence of two well-supported clades (a pre-2007 and post-2007 clade), which was consistent with the clustering pattern of Bayesian inference phylogeny. The statistically significant test of sequence statistics (*K*_ST_ = 0.1604; *P < *0.0001), rank statistics (*Z *= 11812.6132; *P < *0.0001), and nearest neighbor statistics (*S*_nn_ = 1.0000; *P < *0.0001) indicated a strong genetic differentiation between clades I and II. Analysis of molecular variance (AMOVA) results revealed the majority of genetic variation was between clades I and II, accounting for 56.08% of the total variation (*Φ*_ST_ = 0.5608; *P < *0.0001) (see Table S3 in the supplemental material). The very great *F*_ST_ value (*F*_ST_ = 0.5608; *P < *0.0001) suggested that clades I and II were highly genetically differentiated. Collectively, these results demonstrated that clade I was genetically distinct from clade II.

**FIG 2:**
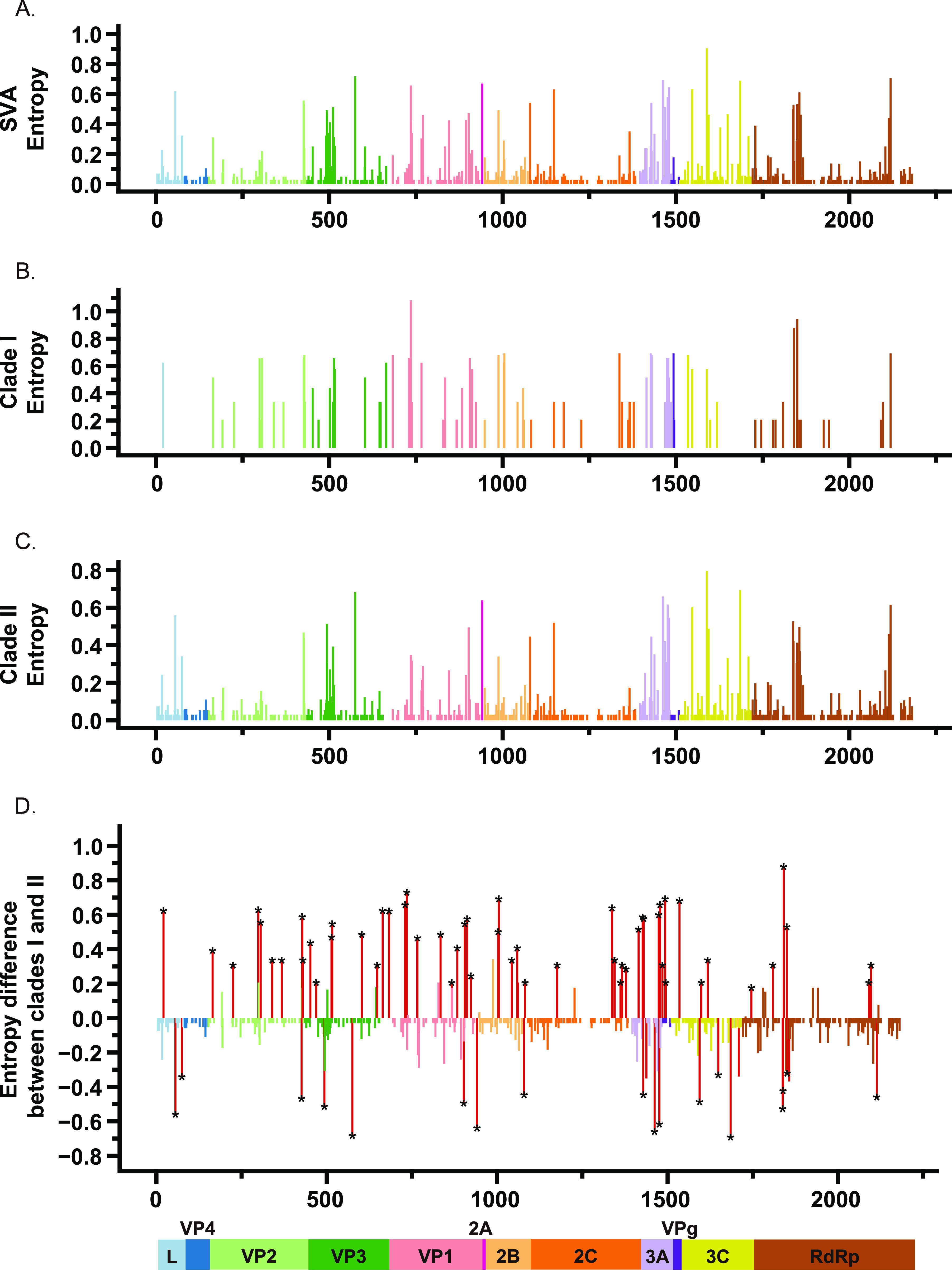
Protein sequence variability of SVA strains. The bars indicate the entropy score of each amino acid in the alignment of all SVA sequences (A), clade I (B), and clade II (C). (D) Entropy difference between sequences from clades I and II. The length of each bar quantifies the entropy difference between clades I and II (clade I in the upper part of the graph and clade II in the lower part). Red colored bars (marked with an asterisk) indicate positions with statistically significant (*P* ≤ 0.05) difference between entropies of the two data sets.

In total, 46 and 498 sites of specific amino acid substitution were identified in clade I and II, respectively (see Table S4 in the supplemental material). Among them, 20 and 159 sites of specific amino acid substitution were located in the capsid proteins (VP1, VP2, VP3, and VP4) of clade I and II, respectively. To identify the underlying sequence signature patterns, we analyzed the entropy of each amino acid position in the sequence alignments of SVA. Shannon entropy analysis revealed a set of highly variable amino acid positions with relatively high entropy in SVA sequences (Fig. 2A). VP3, VP1, 2B, 2C, 3A, 3C, and RdRp proteins had relatively more highly diverse loci compared with other proteins (Fig. 2A). We found that clades I and II had different levels of variability in different amino acid sites (Fig. 2B and 2C). A total of 77 amino acid sites across SVA polyprotein displayed statistically significant differential Shannon entropy (*P *≤ 0.05) between clades I and II (see Table S5 in the supplemental material), of which 37 sites had higher entropy in clade I, while 40 sites had higher entropy in clade II. Twenty-nine amino acid sites of SVA major antigenic proteins (VP1, VP2, and VP3) were identified as having significant entropy differences between clades I and II (Fig. 2D), indicating the antigenic properties between clades I and II were substantially different. In addition, other amino acid sites with significant entropy differences between clades I and II were mainly located on RdRp (11 sites), 3A (10 sites), 2C (9 sites), 3C (7 sites), and 2B (5 sites) proteins, suggesting that the functions and activities of these proteins of the two clades might be different. Overall, the results of the entropy analysis revealed differences in the sequence signature patterns between clades I and II in antigen and functional proteins.

### Selection drove the evolution of SVA.

The results of the branch-site unrestricted test for episodic diversification (BUSTED) showed a highly significant result (*P *< 0.01) in the likelihood ratio test, providing robust evidence that SVA experienced episodic positive selection. Our RELAX analyses inferred a relaxation parameter (*K* = 0.87; *P = *0.0074) indicating that clade II was evolving under relaxed selection compared with clade I. The analyses of single-likelihood ancestor counting (SLAC) and fixed effects likelihood (FEL) identified that the majority of SVA codons were negatively selected (see Table S6A and B in the supplemental material). Evidence provided by SLAC, FEL, fast unconstrained Bayesian approximation (FUBAR), and mixed effects model of evolution (MEME) revealed 27 positively selected codons (Table 1), which spread throughout the SVA genome except in genes of L, 3A, and 3B. Clades I and II had 5 and 24 positive selection sites containing clade-specific amino acid residues, respectively. Of the 27 positively selected sites, 10 and 17 sites were located on the structural and nonstructural protein genes, respectively. The gene with the largest number of positively selected sites was RdRp (sites 54, 141, 241, 243, 369, 400, and 442), followed by VP1 (sites 62, 63, 93, 97, and 194), 3C (sites 16, 20, 56, 68, and 115), 2C (sites 132, 153, and 270), VP4 (sites 5 and 7), VP2 (sites 156 and 277), VP3 (site 41), 2A (site 4), and 2B (site 3).

**TABLE 1:** Summary of positive selection in SVA polyprotein

Polyprotein site	Protein site	Protein name	Selection model(s)	Clade I residues	Clade II residues
84	5	VP4	MEME	T	F/T
86	7	VP4	MEME	S	L/S
306	156	VP2	FEL, MEME	K/N	K/N/S
427	277	VP2	FEL, SLAC, FUBAR, MEME	M/T	K/M/R/T
475	41	VP3	MEME	I	I/V/X
735	62	VP1	FEL, SLAC, FUBAR, MEME	K/L/Q	A/E/K/Q/T/V
736	63	VP1	MEME	E	A/E/T
766	93	VP1	FEL, MEME	A/V	A/V
770	97	VP1	FEL, SLAC, FUBAR, MEME	G	A/D/G
867	194	VP1	MEME	A/R	R
941	4	2A	SLAC	I	I/V/X
949	3	2B	FEL	S	F/S/T
1206	132	2C	MEME	A	A/L
1227	153	2C	MEME	L/S/X	F/L
1344	270	2C	MEME	I/L	I
1524	16	3C	MEME	V	I/V
1528	20	3C	MEME	I	H/I
1564	56	3C	FEL	T	A/I/T
1576	68	3C	MEME	D	D/T
1623	115	3C	MEME	V	C/V
1773	54	RdRp	MEME	V	A/M/V
1860	141	RdRp	MEME	E/V	I/L/M/T/V
1960	241	RdRp	MEME	F	F/N
1962	243	RdRp	MEME	S	L/S
2088	369	RdRp	MEME	V	A/I/V
2119	400	RdRp	FEL, SLAC, FUBAR, MEME	L/S	A/L/S/V
2161	442	RdRp	FEL	A	A/V

**FIG 3:**
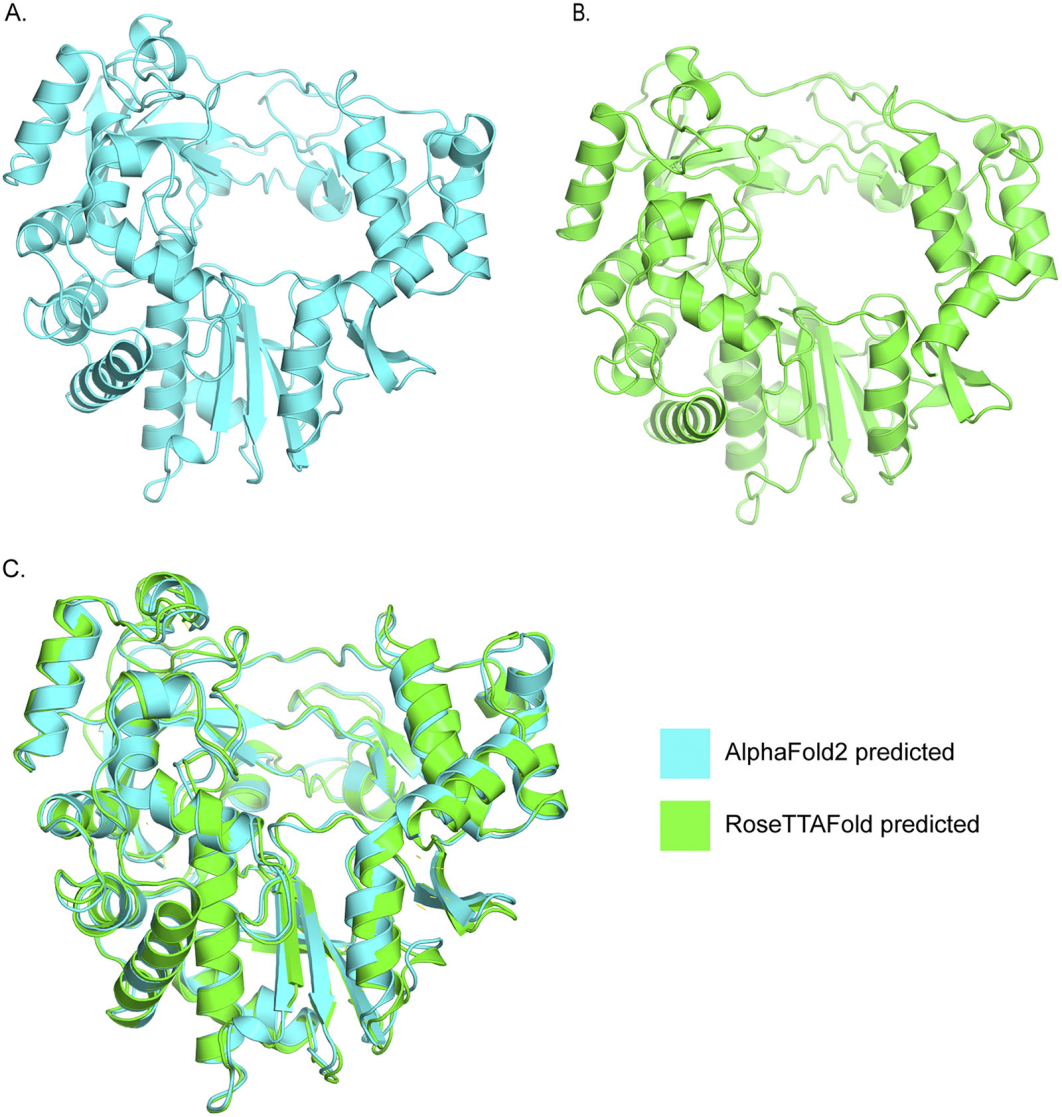
Superposition of RdRp structures predicted by RoseTTAFold and AlphaFold2. (A) The RdRp structure predicted by AlphaFold2. (B) The RdRp structure predicted by RoseTTAFold. (C) Superposition of RdRp structures predicted by RoseTTAFold and AlphaFold2. The green and cyan indicate the RdRp structures predicted by RoseTTAFold and AlphaFold2, respectively.

**FIG 4:**
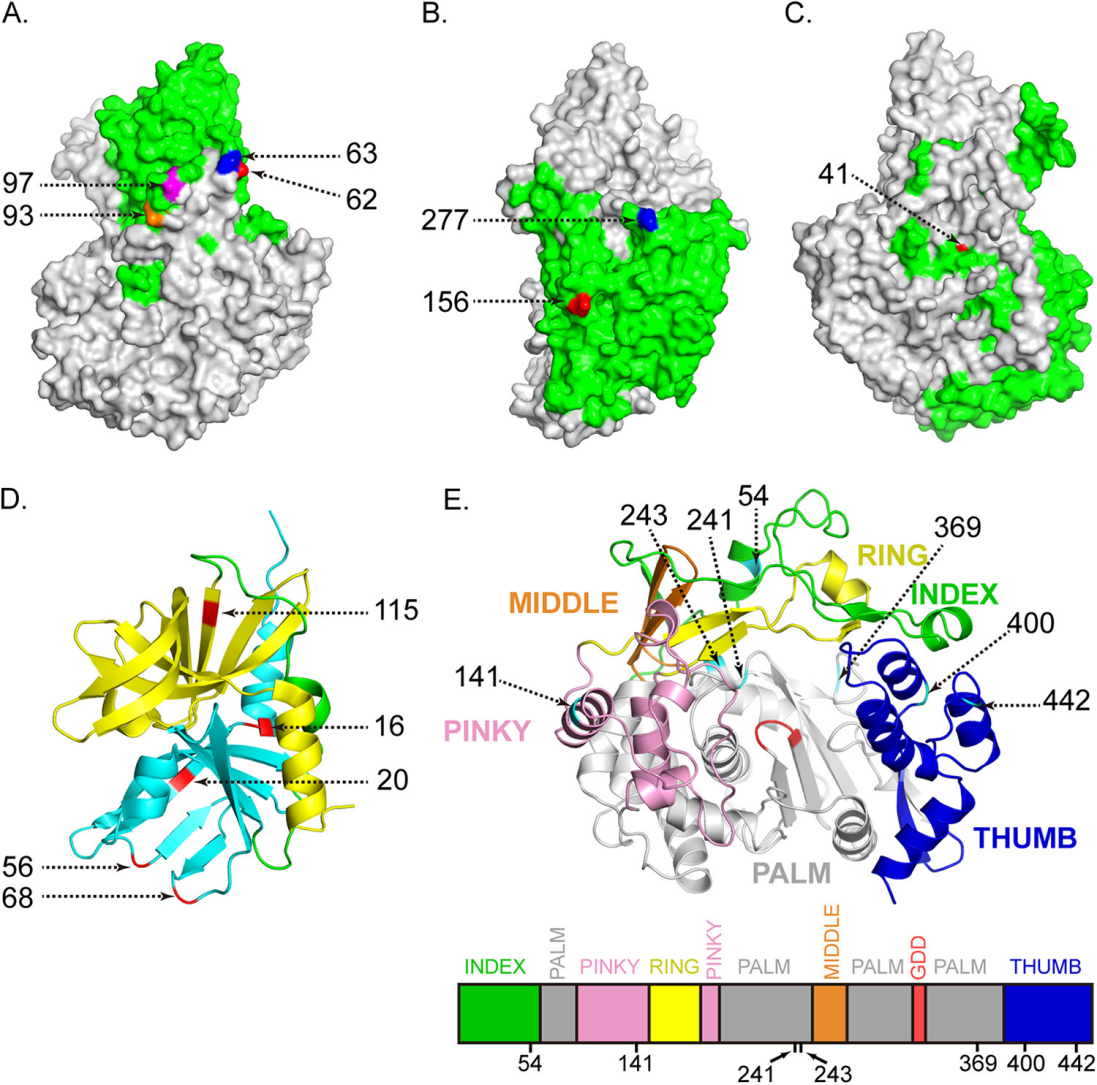
Mapping of the positively selected amino acids on the structural model of VPs, 3C, and RdRp. (A) Localization of positively selected residues on the outer surface of VP1 protein. The green indicates VP1 protein exposed on the outer surface of capsid protein protomer. The positively selected residues 62, 63, 93, and 97 are colored in red, blue, orange, and magenta, respectively. (B) Localization of positively selected residues on the outer surface of VP2 protein. The green indicates VP2 protein exposed on the outer surface of capsid protein protomer. The positively selected residues 156 and 277 are colored in red and blue, respectively. (C) Localization of positively selected residues on the outer surface of VP3 protein. The green indicates VP3 protein exposed on the outer surface of capsid protein protomer. The positively selected residue 41 is colored in red. (D) Localization of positively selected residues on 3C protease. The yellow, cyan, and green indicate the first (N-terminal) domain, second (C-terminal) domain, and connecting loop, respectively. The positively selected residues 16, 20, 56, 68, and 115 are colored in red. (E) Top view of RdRp structure highlighting positively selected residues. The RdRp structure was predicted using AlphaFold2. The (sub)domain distribution is shown as follows: fingers with colored fingertips (index in green, middle in orange, ring in yellow, and pinky in pink), palm in gray, thumb in blue. The conserved GDD (Gly-Asp-Asp) catalytic active site is colored in red. The positively selected residues 54, 141, 241, 243, 369, 400, and 442 are colored in cyan. Below bar represents the RdRp sequence colored according to the structural elements mentioned above.

The calculated root mean square deviation (RMSD) value between AlphaFold2-predicted and RoseTTAFold-predicted RdRp structures was 0.800, suggesting the RdRp structures predicted by the two methods were identical (Fig. 3). Structural analysis showed that seven positively selected sites (sites 62, 63, 93, and 97 of VP1 protein, sites 156 and 277 of VP2 protein, and site 41 of VP3 protein) were exposed on the outer surface of the capsid protein protomer. One (site 115) and four (sites 16, 20, 56, and 68) positive selection codons were located on the C-terminal and N-terminal domains of the 3C protease, respectively (Fig. 4). The positively selected sites of the RdRp protein were distributed among multidomains, including the index finger domain (site 54), pinky finger domain (site 141), palm domain (sites 241, 243, and 369), and thumb domain (sites 400 and 442) (Fig. 4). These results demonstrated that both positive selection and negative selection together drove the evolution of SVA.

### Evolutionary dynamics of SVA.

TempEst analysis did not find the potential problematic sequences from the data set. The analysis of Bayesian evaluation of temporal signal (BETS) revealed that SVA evolution was characterized by a very strong temporal signal (Bayes factor [BF] = 759.30), indicating a strongly clock-like evolution of SVA. The identified best-fit model combination was the general time-reversible (GTR) model with fixed empirical base frequencies (F) and invariable sites (I) and gamma-distributed evolutionary rates (G) (number of gamma parameters, 4) (GTR+F+I+G4), uncorrelated relaxed log-normal (UCLN) clock model, and coalescent Bayesian SkyGrid model.

**FIG 5:**
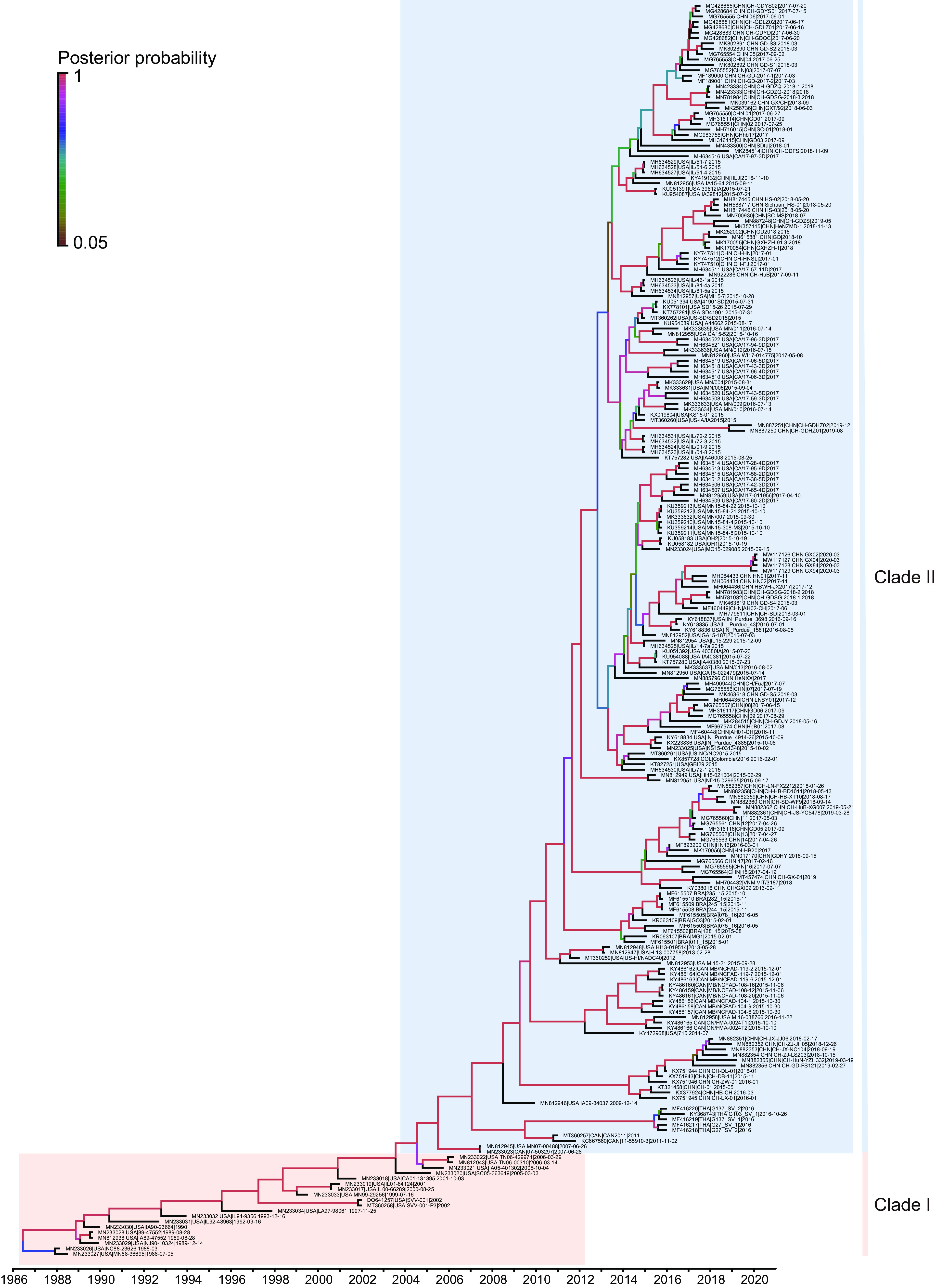
Time-scaled maximum clade credibility tree of SVA strains. The Bayesian phylogenetic tree was inferred using the relaxed molecular clock and Bayesian SkyGrid model. The branch color represents the posterior probability as indicated by the scale in the left region of the figure. Horizontal axis indicates chronological time, expressed in years. The two main clades are indicated using the color blocks. The red block represents clade I isolated pre-2007. The blue block represents clade II isolated post-2007.

**FIG 6:**
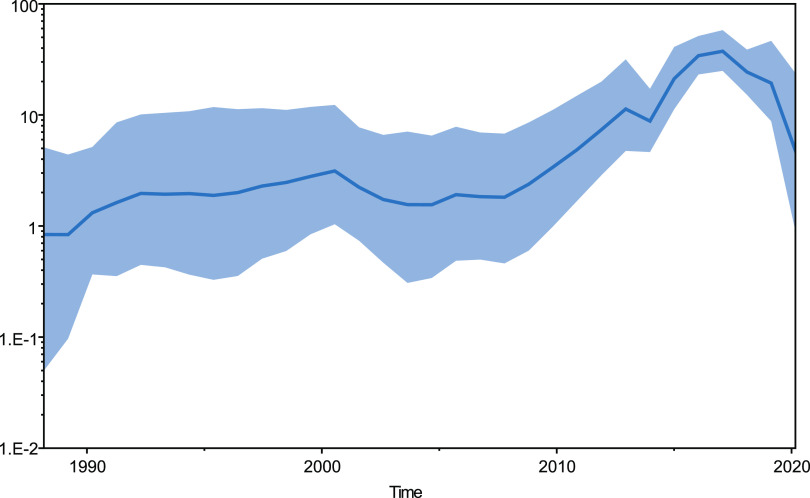
Bayesian SkyGrid plot illustrating temporal changes in the effective population size of SVA strains. The *y* axis indicates the effective population size of SVA strains. The *x* axis represents the timeline. Line plots summarize estimates of the effective population size, a measure of genetic diversity. The dark blue line shows the mean value of the effective population size, and the light blue shading shows the 95% HPD.

The maximum clade credibility (MCC) tree topology of SVA genome was characterized by the short terminal branches (Fig. 5). The estimated mean rate of evolution over the genome was 3.3522 × 10^−3^ nucleotide substitutions/site/year (subs/site/year) (95% highest posterior density [HPD], 2.9399 × 10^−3^ to 3.7668 × 10^−3^ subs/site/year). Correspondingly, the estimated time to the most recent common ancestor (TMRCA) of SVA was dated to 1986 (95% HPD, 1983.9138 to 1988.1639). The TMRCAs of SVA clade I and clade II were estimated as 1986 (95% HPD, 1983.605 to 1988.1639) and 2004 (95% HPD, 2000.8054 to 2007.4822), respectively. Viral effective population size displayed a gradually increasing trend with slight fluctuation from the TMRCA until 2017, followed by a sharp decline (Fig. 6).

### The source location and spread routes of SVA since 2015.

**FIG 7:**
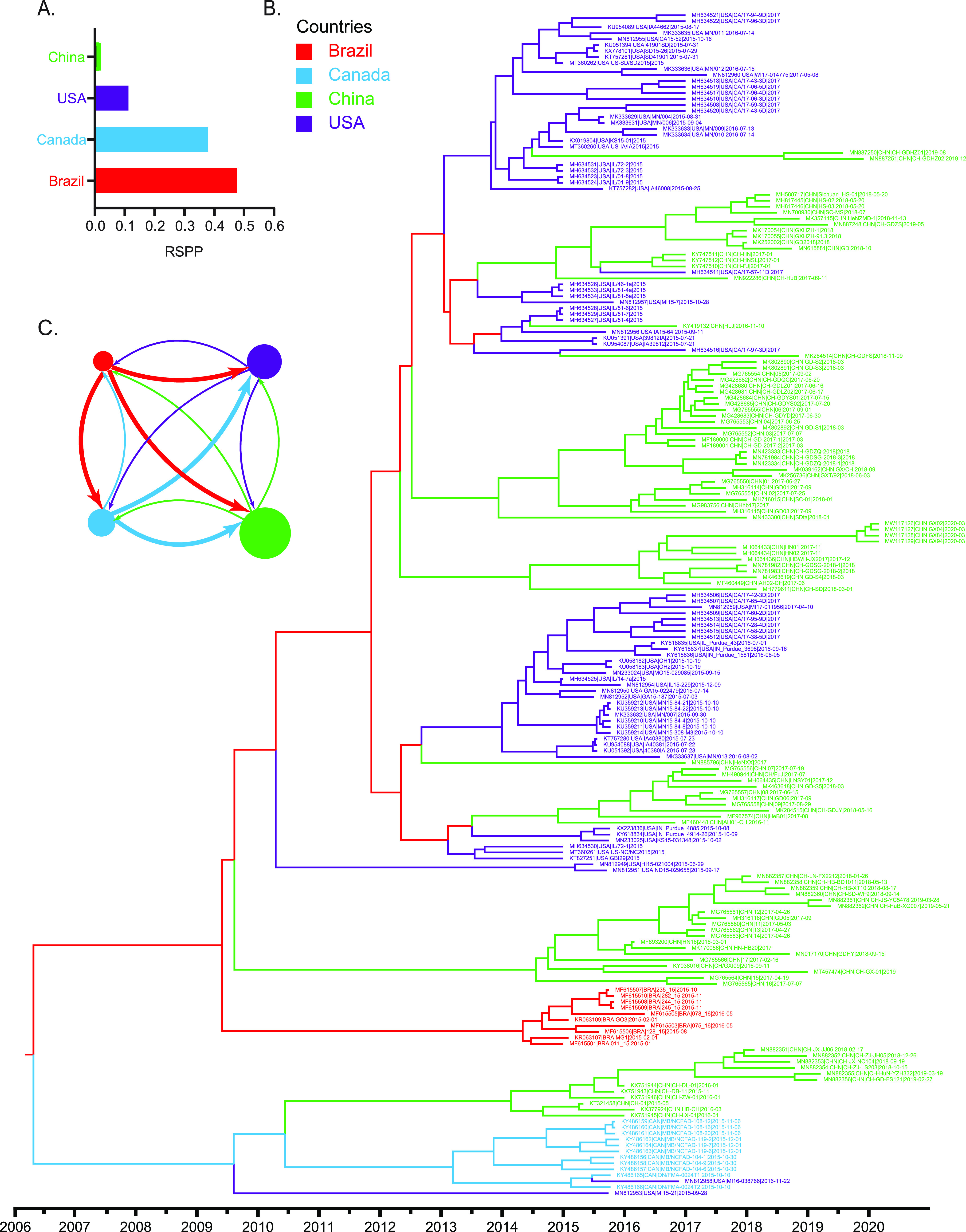
The marginal approximation of the structured coalescent (MASCOT) analysis of SVA global outbreak since 2015. (A) The root state posterior probabilities for each country estimated by MASCOT model. Brazil is inferred to be with the highest posterior probability support (RSPP = 0.4811). (B) Structured coalescent phylogeographic analysis of SVA strains using MASCOT model. The branch color indicates the inferred ancestral location with the largest posterior probability. Color changes in the branch indicate virus migration events. MASCOT analysis places the source of SVA’s global transmission since 2015 in Brazil, from where it spread to Canada, China, and the United States. (C) The effective population size (circles) and immigration rates (arrows) estimated by the MASCOT model. Circles are colored based on the countries of SVA outbreak since 2015. The circle sizes indicate the inferred effective population sizes of the countries. The different source countries are denoted by arrow colors. The width of arrow indicates the median inferred immigration rates. The wider arrow into a country, the more likely it is that virus in the destination country originated from the source country of that arrow.

**FIG 8:**
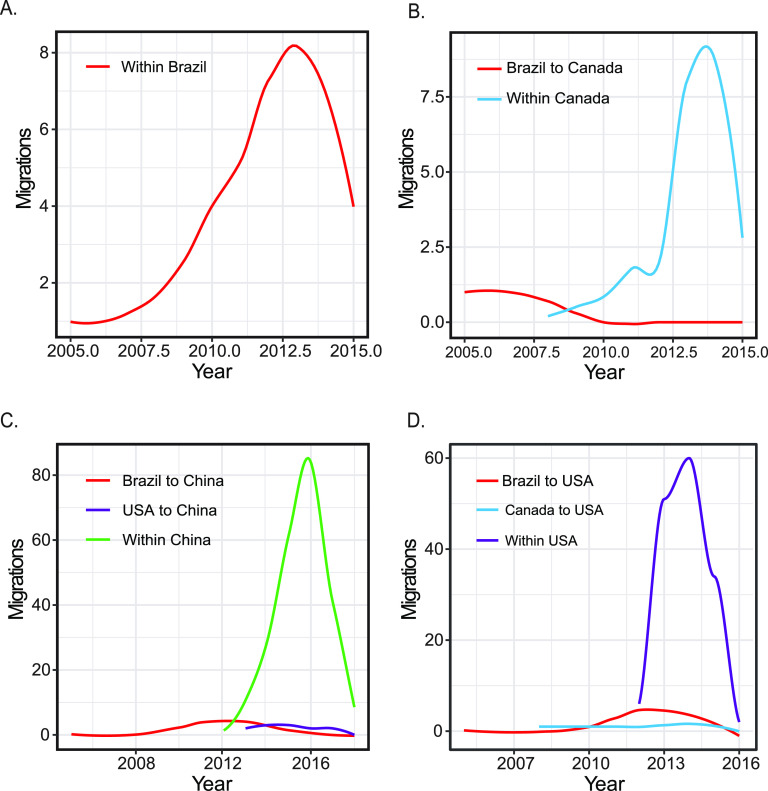
Migrations (on log_10_ scale) of SVA through time. The plots show within-country migration/transmission in Brazil (A), Canada (B), China (C), and the United States (D) over time. The plots also show the relative importance of SVA immigration for within-country migration/transmission.

The spatial circulation of SVA between different countries since 2015 was inferred by phylogeographical analysis. Our phylogenetic analysis placed the root location of the MCC tree in Brazil with the highest posterior probability support (root state posterior probabilities [RSPP] = 0.4811), suggesting Brazil was inferred as the most likely source of SVA outbreaks since 2015 (Fig. 7A and 7B). Furthermore, the estimated RSPP (0.4811) is outside the range of probabilities (0.2039 to 0.2846) obtained in the location randomized analyses of 20 replicate data sets (see Fig. S3 in the supplemental material), providing further support for the reliability of Brazil at the root node. China was estimated to have the largest effective population size, followed by the United States, Canada, and Brazil, suggesting that SVA lineages in China had the largest genetic diversity compared to the other countries. Most interestingly, we found that SVA spread since 2015 displayed a strongly asymmetric diffusion pattern (Fig. 7C). The asymmetric diffusion model, also known as the source-sink model, allows directionality to be inferred so that each viral transition is characterized by a source (i.e., origin of the virus) and a sink (i.e., destination) (28). The asymmetric pattern of SVA diffusion indicated that SVA spread since 2015 was directional and characterized by geographical sources and destinations. Brazil was deduced to be a main source for strains isolated since 2015 in Canada, the United States, and China. Strains isolated from the United States since 2015 were inferred to be mainly from Brazil and Canada, while strains isolated from China since 2015 were mainly from Brazil and the United States. Interestingly, the internal transmissions of SVA in the United States, Canada, and China became dominant when SVA immigrated (Fig. 8). These results revealed that Brazil may play a decisive role in seeding SVA epidemics since 2015.

## DISCUSSION

Although SVA was discovered 2 decades ago, the detailed evolutionary characteristics of SVA remain elusive. Here, we have generated the most robust phylogeny of SVA to date by substantially using all publicly published data and applying comprehensive analytical methods to study the evolution of SVA. Notably, our phylogenetic analyses revealed that SVA might have originated in 1986 and has differentiated into two branches: clades I (pre-2007 strains) and II (post-2007 strains). Remarkably, the signals of positive and negative selections spread throughout SVA genome. Most importantly, our phylogeographic analyses suggested Brazil might be the source location of SVA outbreak since 2015.

Comparatively speaking, recombination appears to occur at a high frequency in picornaviruses (29) and readily occurs between closely related strains (intratypic recombination) in picornaviruses (30). Previous studies successfully identified one and eight recombinants isolated from Canada (31) and China (32–35), respectively. Unlike previous reports, our rigorous analysis only identified five recombinants, all of which were isolated from China. Meanwhile, our MASCOT results showed that the SVA population in China had the largest effective population size, which was conducive to SVA recombination.

It has been reported that SVA may be classified into one (22), two (3, 23–25), or three (26, 27) main clades. In this study, we performed rigorous phylogenetic tree-building of SVA using all publicly published genomes. Our BI tree revealed two distinct and well-supported clades of SVA, clades I (pre-2007 strains) and II (post-2007 strains). Meanwhile, our study identified a very high degree of genetic differentiation (*F*_ST_ = 0.5608) between clade I and clade II. Although SVA had been spread in the swine population since at least 1988 (8), SVA-caused vesicular disease was firstly identified in 2007 (9). Furthermore, pigs inoculated with strains of clade I (based on our definition) didn’t show specific clinical disease (8, 36, 37), whereas pig infected by strains of clade II (according to our definition) resulted in characteristic clinical signs and vesicular lesions (4, 36, 38, 39). Taken together with our results and this mounting body of experimental evidence, it is reasonable to conclude that SVA underwent a major genetic differentiation in 2007, creating a new evolutionary clade and leading to virulence enhancement.

The present study revealed that 27 sites of SVA polyprotein were under strong positive selection, findings which differed from previous study (23). It is worth noting that 24 positive selection sites of clade II contained the clade-specific amino acid residues. The clade II-specific amino acid substitutions under positive selection could be involved in the mechanism of SVA clade replacement and pathogenicity. In this study, the most positive selection sites localized in the genes of capsid proteins (VP1 to VP4), 3C protease, and RdRp. The structural proteins (VP1 to VP4) form the viral capsid, which is responsible for receptor recognition (40) and immune escape (41). The protruding loop structures of the SVA capsid surface involve receptor binding and cell tropism, and contain B cell epitopes (25, 42). As the cellular receptor of SVA, anthrax toxin receptor 1 (ANTXR1) engages the surface-exposed BC loop and loop II of VP1 protein and “the puff” loop of VP2 protein (40). Interestingly, our analysis showed that positively selected sites are located in the BC loop (sites 62 and 63), loop II (sites 93 and 97), and GH loop (site 194) of the VP1 gene and in “the puff” loop (site 156) of the VP2 gene, suggesting that they were likely to play a key role in receptor recognition and immune escape. In view of the important role of the 3C protease in antagonizing the production of type I interferon (43), inducing apoptosis (44), and promoting viral replication (45), five positively selected codons of 3C protease found in this study may play important roles in regulating the function of 3C protease. It is worth noting that seven positively selected codons identified in our study distributed the index finger domain, pinky finger domain, palm domain, and thumb domain of RdRp, suggesting a vital role in helping SVA fine tune replication fidelity and quasispecies distributions. Further studies are needed to elucidate the roles of these positive selection sites in receptor binding, cell entry, transcription, proliferation, innate immune evasion, virulence, and pathogenesis, which are the base of viral adaptive evolution.

In this study, the estimated evolutionary rate of SVA was 3.3522 × 10^−3^ subs/site/year (95% HPD, 2.9399 × 10^−3^ to 3.7668 × 10^−3^ subs/site/year), which was much higher than the previously reported 2.696 × 10^−3^ subs/site/year (95% HPD, 2.505 × 10^−3^ to 2.905 × 10^−3^ subs/site/year) (24). The estimated TMRCA of SVA was around 1986 (95% HPD, 1983 to 1988), which corresponded to previous findings that SVA has been present in pig herds since at least 1988 (8). The ladder-like topology was observed for clade I strains collected in the United States before 2007, which could be caused by step-by-step transmission of the virus in swine in the United States, while the tree topology of SVA clade II was caused by the epidemic outbreak of SVA-associated vesicular disease since 2015. In line with the trend of SVA infection, our analysis revealed a distinct increase in the effective population size until 2017 followed by a dramatic decline. In view of the high titers of anti-SVA neutralizing antibodies in naturally infected pigs (46), the sharp drop in the effective population size of SVA after 2017 was likely caused by the herd immunity of the pig population, which was acquired after the concentrated outbreak of SVA-associated vesicular disease in 2015 and 2016.

Using discrete phylogeographic reconstruction, we first explored the historical migration routes of SVA pandemic since 2015 among Brazil, the United States, Canada, and China. Our study showed that Brazil was inferred to be the location of geographical origin and the source of global transmission of SVA-associated vesicular disease since 2015. Considering the geographic isolation of these countries, SVA might be transmitted via the transboundary trades of contaminated live pigs or feed (47–51). From the perspective of geographic transmission, we suggest that an extensive and in-depth surveillance of transboundary trades in live pigs, feed, and related ingredients will help control the global outbreaks of SVA-associated vesicular disease.

In conclusion, our phylogenetic analysis demonstrated that SVA diverged into two main branches, clade I (pre-2007 strains) and clade II (post-2007 strains). Selection pressure analysis showed clade II was under less selective pressure than clade I, and both negative and positive selections were the important driving forces for SVA evolution. Phylodynamic analysis suggested that the evolutionary rate of SVA was 3.3522 × 10^−3^ subs/site/year, and the TMRCA of SVA was around 1986. Demographic history analysis revealed that the effective population size of SVA displayed a gradually increasing trend with slight fluctuation until 2017 followed by a sharp decline. Phylogeographic analysis found that Brazil might be the source location of SVA’s global spread since 2015. Our study provides comprehensive insights into the recombination, evolutionary history, spatiotemporal dynamics, and selection pressures of SVA.

## MATERIALS AND METHODS

### Ethics statement.

All animal care and experimental procedures were approved by the Institutional Animal Care and Use Committee (IACUC) of Yangzhou University. The IACUC approval numbers were 201709012, 201803191, and 202003411.

### Sequence data.

Complete SVA genome sequences were downloaded from the GenBank database of the National Center for Biotechnology Information (NCBI) until December 2021. Detailed sequence information (accession number, strain name, location, isolation year) for 249 complete genome sequences of SVA are available in the supplementary material (see Table S1 in the supplemental material). For genomes having identical CDS, we retained only one out of several genomes. All CDSs were extracted and aligned using the codon-based aligner MACSE (Multiple Alignment of Coding SEquences) (version 2.03) (52, 53).

### Recombination detection.

Considering that recombination affects phylogeny estimation, the potential mosaic recombinant structures of SVA genomes were screened using the strategy as below. The aligned sequences were examined using the RDP4 (version 4.101) (54) with default configuration for seven different recombination detection algorithms, namely, RDP, GENECONV, Chimeara, MaxChi, BootScan, 3Seq, and SiSca. In this study, only those sequences with events that were significant (*P *< 1.0E−6) by at least four algorithms were considered as recombinants. To further validate the results of RDP4, the sequence similarity plots were carried out using the seqcombo package (version 1.14.1) (55) of the R program (version 4.1.0) (56). The recombinant sequences were removed from the data set. The remaining sequences were aligned, and the recombination signals were detected again with the criteria mentioned above until there was no recombination signal. The recombinant sequences were removed from any further analyses of this study.

### Phylogeny analysis.

The complete data set without recombinant sequences was assessed for strength of phylogenetic signal by applying the likelihood mapping approach implemented in IQ-TREE (version 1.6.12) (57). The nucleotide substitution saturation was estimated using Xia’s test (58) as implemented in the DAMBE (version 7.3.5) (59). Xia’s test compares an observed index of saturation substitution (*I*_ss_) to a critical index of substitution saturation (*I*_ss.c_). If *I*_ss_ is significantly less than *I*_ss.c_, data will be considered as little saturation in transition and transversion, and data are good for phylogenetic analysis (58).

GTR+F+I+G4 was chosen as the best fitting model based on Bayesian information criterion (BIC) using the ModelFinder (60) module in IQ-TREE (version 1.6.12) (57). The Bayesian inference (BI) approach was used to reconstruct the phylogenetic relationships of SVA strains implemented in MrBayes (61) (version 3.2.7a). The Markov chain was run for 10 million generations with a sampling parameter every 1,000 generations, and the first 25% of Markov chain Monte Carlo (MCMC) samples were discarded as burn-in. The topology convergence of BI trees was verified by the average standard deviation of split frequencies (ASDSF) (<0.001) (62). Chain stationarity was confirmed based on the raw trace obtained in Tracer (version 1.7.1) (63). The convergence of parameters was assessed by the average potential scale reduction factor (PSRF) (close to 1) and effective sample sizes (ESS) (>200).

### Estimation of genetic differentiation.

We calculated the genetic distance between two clades of SVA. The pairwise genetic distance between two clades was estimated using MEGA (version 11) (64) with the maximum composite likelihood model. Variance was estimated using 1,000 bootstrap replications, and the rate variation among sites was modeled with a gamma distribution (shape parameter = 1). The two-sample *t* test was performed by DIVEIN (65) to compare the means of genetic sequence diversity between two clades.

We assessed SVA clades using PhyloPart (version 2.1) (66), a methodology of phylogeny partition. For PhyloPart analysis, the optimal percentile threshold assignment is determined by investigating the percentile threshold range of the pairwise patristic distance distribution. The phylogenetic tree was divided into clusters based on criteria of >90 bootstrap branch support and the optimal percentile threshold (49.6%).

To determine the presence of differentiation in SVA isolates, the genetic differentiation parameters, *viz.*, the sequence statistic (*K*_ST_), the rank statistic (*Z*), and the nearest-neighbor statistic (*S*_nn_), were estimated using DnaSP (67) (version 6.12.03). To assess the contribution of various factors to the genetic differentiation of SVA clades, analysis of the molecular variance (AMOVA) was performed with Arlequin (68) (version 3.5.2.2), and 10,000 permutations were conducted to evaluate the significance of factors. *F*_ST_-based genetic distances were estimated by Arlequin (68) (version 3.5.2.2). Significant deviations from null clade differentiation were tested by performing 10,000 permutations. The degree of genetic differentiation was assessed using the ranges of *F*_ST_ specified as follows (69): 0 < *F*_ST_ ≤ 0.05, little differentiation; 0.05 < *F*_ST_ ≤ 0.15, moderate differentiation; 0.15 < *F*_ST_ ≤ 0.25, great differentiation; *F*_ST_ > 0.25, very great differentiation.

### Measure of variability.

The clade-specific amino acid substitutions of clades I and II were identified. Shannon entropy was used as a measure of the protein sequence variability at each position in data sets of SVA strains. Shannon entropy is a quantitative measure of variability, where higher entropy means higher variability (70). Statistical significance in observed entropy differences between clades I and II at each amino acid position were evaluated using the Monte Carlo randomization method (5 out of 100; with replacements) with Bonferroni correction (70).

### Selection pressure analysis.

To test if some sites of the SVA genome have been subject to positive diversifying selection, we performed the branch-site unrestricted test for episodic diversification (BUSTED) (71) implemented in the program of hypothesis testing using phylogenies (HyPhy) (72) (version 2.5.31). We investigated selective differences between clades I and II using RELAX (73) of HyPhy (72) (version 2.5.31). Furthermore, the sites under pervasive positive selection were identified using four different site-specific methods in HyPhy (72) (version 2.5.31) as follows: single-likelihood ancestor counting (SLAC) (74), fixed effects likelihood (FEL) (74), fast unconstrained Bayesian approximation (FUBAR) (75), and mixed effects model of evolution (MEME) (76). Sites with a *P *value of <0.1 for SLAC, FEL, and MEME, and sites with a posterior probability of >0.9 for FUBAR were considered statistically significant for selection.

### Protein structure analysis of adaptive evolution sites.

The structures of SVA capsid protomer (complex of VP1, VP2, VP3, and VP4) and 3C protease have already been experimentally determined (77). However, no such information is available for RdRp of SVA. The theoretical three-dimensional structure for RdRp was generated using two deep-learning algorithms, AlphaFold2 (78) and RoseTTAFold (79). For the AlphaFold2 approach, the amino acid sequence of RdRp was submitted to ColabFold (80), which accelerated protein structure prediction by combining the fast homology search of MMseqs2 with AlphaFold2. For the RoseTTAFold method, the amino acid sequence of RdRp was submitted to the online RoseTTAFold server (https://robetta.bakerlab.org/). The protein structures were depicted in PyMOL (81) (version open-source build 2.5.0). The root mean square deviation (RMSD) value calculated by PyMOL was used to measure the structural similarity between the AlphaFold2-predicted and RoseTTAFold-predicted protein structures. Proteins with an RMSD value of less than 3.0 Å were considered to have the same structure (82). The locations of positively selected sites were depicted on the crystal structure of the capsid protein protomer (PDB accession number 6ADT), 3C protease (PDB accession number 6L0T), and the predicted structure of RdRp using PyMOL (81) (version open-source build 2.5.0).

### Data exploration and test of temporal signal.

To identify potential problematic sequences resulting from sample contamination or mislabeling, a regression graph of the root-to-tip distances against the sampling times was plotted using TempEst (83) (version 1.5.1). The temporal signal of data set was assessed using the Bayesian evaluation of temporal signal (84) (BETS). BETS compares the fit of the original heterochronous model (M_het_) (with original sampling times) and the corresponding isochronous model (M_iso_) (with a fixed rate). Marginal likelihood estimation (MLE) was performed using generalized stepping-stone sampling (85). Parameters were estimated using the MCMC approach implemented in BEAST (version 1.10.4) (86). MCMC chains were run for 200 million steps and sampled every 20,000 steps. The BEAGLE (87) library (version 3.1.0) was used to perform massive parallelization on computing architectures. A (log) Bayes factor log(P(Y|M_het_)) − log(P(Y|M_iso_)) of at least 5 indicates a strong temporal signal in the data set (84).

### Phylodynamics analysis.

The best-fit nucleotide substitution model was selected using the ModelFinder (60) module in IQ-TREE (version 1.6.12) (57). The best-fit model combinations of molecular clock models (strict clock and uncorrelated relaxed log-normal [UCLN] clock models) and coalescent demographic models (constant population size, exponential growth, Bayesian SkyGrid, and Bayesian Skyline) were determined by the path sampling and stepping-stone sampling (PS/SS) methods (88). The combinations of molecular clock models and coalescent models were compared using marginal likelihoods. The nucleotide substitution rate and the TMRCA of SVA were estimated through the MCMC framework implemented in BEAST (version 1.10.4) (86). Five independent Bayesian analyses were run for 500 million MCMC steps with sampling parameters and trees every 50,000 steps. The BEAGLE (87) (version 3.1.0) library program was used for computational enhancement. Convergence of parameters was evaluated using Tracer (63) (version 1.7.1) with 10% burn-in of the total chain length. ESS values of parameters greater than 200 were considered acceptable. The estimated clock rate and TMRCA were extracted using Tracer (63) (version 1.7.1). All tree files were combined by LogCombiner (version 2.6.6), a package of BEAST (version 2.6.6) (89). The MCC tree was summarized from the entire sample of trees using TreeAnnotator (version 1.10.4) (90) after discarding the first 10% as burn-in. MCC tree was visualized with FigTree (version 1.4.4) (91).

### Phylogeographic analysis.

To explore the historical migration routes of SVA pandemics since 2015, the phylogeographical inference was performed using the marginal approximation of the structured coalescent (MASCOT) (92) package (version 2.1.2) implemented in BEAST (version 2.6.6) (89). The MASCOT approach reconstructs the evolutionary tree while considering the sizes of different subpopulations and improves the inference accuracy of the migration rates between spatial demes (92). The migration rates, effective population sizes, and locations of internal nodes were also estimated by the MASCOT package. The genomic data set was analyzed with the model combination of UCLN clock model and GTR+F+I+G4 nucleotide substitution model. Three independent MASCOT analyses were run for 200 million iterations with sampling parameters and trees every 20,000 iterations. Convergence of parameters was checked by Tracer (63) (version 1.7.1) with 10% burn-in of the total chain length. All tree files were merged by LogCombiner (version 2.6.6) (89). TreeAnnotator (version 1.10.4) (90) was used to summarize the MCC tree after discarding the first 10% as burn-in. FigTree (version 1.4.4) (91) was employed to visualize the MCC tree. The root state posterior probabilities (RSPP) for the ancestral geographic states were also summarized from the posterior density of trees by TreeAnnotator (version 1.10.4) (90). To assess the reliability of the inferred location at the root node, we carried out the location randomization analysis. We produced 20 replicate data sets, in which the location status was randomly swapped among the sequences. Each of these data sets was analyzed using Bayesian phylogeographic method as described above.

### SVA migration over time.

The migration patterns of SVA over time were estimated using a similar method to that developed by O’Neill et al. (93). The MCC tree generated from the MASCOT analysis was processed using the in-house script (94) (available at GitHub, https://github.com/admiralenola/globall4scripts) under the Python programming framework of Environment for Tree Exploration (ETE) (95) (version 3.1.2). The time tree was traversed in a sliding window fashion. The number of clades corresponding to four different demes were recorded for each year. The migration events were assumed to occur on nodes. This assumption might lead to a slight bias toward inflated ages of migration events, which has a more pronounced impact on the very early migration events but can be negligible for later migrations due to extensive branching (94).

### Data availability.

All relevant data are within the manuscript and its supplemental material.
