# Supplementary material for: The Evolution and Global Spatiotemporal Dynamics of Senecavirus A
Source: Microbiol Spectr. 2022 Oct 31;10(6):e02090-22. doi: 10.1128/spectrum.02090-22 (PMC9769604; doi:10.1128/spectrum.02090-22)
Supplement: Supplemental file 1 — Tables S1, S2, and S3; Fig. S1, S2, and S3. Download spectrum.02090-22-s0001.pdf, PDF file, 1.6 MB [file spectrum.02090-22-s0001.pdf]

Table S1. The detailed information of SVA strains used in this study. Fourteen strains isolated from our laboratory were marked with bold.

Locus	Strain	Country	Collection date
DQ641257	SVV-001	USA	2002
KC667560	11-55910-3	Canada	2-Nov-11
KR063107	SVA/BRA/MG1/2015	Brazil	1-Feb-15
KR063108	SVA/BRA/MG2/2015	Brazil	1-Feb-15
KR063109	SVA/BRA/GO3/2015	Brazil	1-Feb-15
KT757280	USA/IA40380/2015	USA	23-Jul-15
KT757281	USA/SD41901/2015	USA	31-Jul-15
KT757282	USA/IA46008/2015	USA	25-Aug-15
KT321458	CH-01-2015	China	May-15
KU359210	MN15-84-4	USA	10-Oct-15
KU359211	MN15-84-8	USA	10-Oct-15
KU359212	MN15-84-21	USA	10-Oct-15
KU359213	MN15-84-22	USA	10-Oct-15
KU359214	MN15-308-M3	USA	10-Oct-15
KU058182	SVA-OH1	USA	19-Oct-15
KU058183	SVA-OH2	USA	19-Oct-15
KT827251	USA/GBI29/2015	USA	2015
KU051391	US-15-39812IA	USA	21-Jul-15
KU051392	US-15-40380IA	USA	23-Jul-15
KU051393	US-15-40381IA	USA	22-Jul-15
KU051394	US-15-41901SD	USA	31-Jul-15
KX019804	KS15-01	USA	2015
KX377924	HB-CH-2016	China	Mar-16
KX223836	USA/IN_Purdue_4885/2015	USA	8-Oct-15
KX778101	SD15-26	USA	29-Jul-15
KX857728	Colombia/2016	Colombia	Feb-16
KY172968	SVA-715	USA	Jul-14
KU954087	USA/IA39812/2015_P1	USA	21-Jul-15
KU954088	USA/IA40381/2015_P1	USA	22-Jul-15
KU954089	USA/IA44662/2015_P1	USA	17-Aug-15
KU954090	USA/IA44952/2015_P1	USA	17-Aug-15
KX751943	CH-DB-11-2015	China	Nov-15
KX751944	CH-DL-01-2016	China	Jan-16
KX751945	CH-LX-01-2016	China	Jan-16
KX751946	CH-ZW-01-2016	China	Jan-16
KY038016	CH/GXI09/2016	China	11-Sep-16
KY486156	SVA/Canada/MB/NCFAD-104-1/2015	Canada	30-Oct-15
KY486157	SVA/Canada/MB/NCFAD-104-6/2015	Canada	30-Oct-15
KY486158	SVA/Canada/MB/NCFAD-104-9/2015	Canada	30-Oct-15
KY486159	SVA/Canada/MB/NCFAD-108-12/2015	Canada	6-Nov-15
KY486160	SVA/Canada/MB/NCFAD-108-16/2015	Canada	6-Nov-15

Locus	Strain	Country	Collection date
KY486161	SVA/Canada/MB/NCFAD-108-20/2015	Canada	6-Nov-15
KY486162	SVA/Canada/MB/NCFAD-119-2/2015	Canada	1-Dec-15
KY486163	SVA/Canada/MB/NCFAD-119-6/2015	Canada	1-Dec-15
KY486164	SVA/Canada/MB/NCFAD-119-7/2015	Canada	1-Dec-15
KY486165	SVA/Canada/ON/FMA-2015-0024T1/2015	Canada	10-Oct-15
KY486166	SVA/Canada/ON/FMA-2015-0024T2/2015	Canada	10-Oct-15
KY368743	G103_SV_1/2016/Thailand	Thailand	26-Oct-16
KY368744	G103_SV_2/2016/Thailand	Thailand	26-Oct-16
KY747510	CH-FJ-2017	China	Jan-17
KY747511	CH-HN-2017	China	Jan-17
KY747512	CH-HNSL-2017	China	Jan-17
KY419132	SVA/HLJ/CHA/2016	China	2016/11/10
MF893200	SVV HN16	China	1-Mar-16
KY618834	USA/IN_Purdue_4914-26/2015	USA	9-Oct-15
KY618835	USA/IL_Purdue_43/2016	USA	1-Jul-16
KY618836	USA/IN_Purdue_1581/2016	USA	5-Aug-16
KY618837	USA/IN_Purdue_3698/2016	USA	16-Sep-16
MF416217	G27_SV_1/2016/Thailand	Thailand	2016
MF416218	G27_SV_2/2016/Thailand	Thailand	2016
MF416219	G137_SV_1/2016/Thailand	Thailand	2016
MF416220	G137_SV_2/2016/Thailand	Thailand	2016
MF189000	CH-GD-2017-1	China	Mar-17
MF189001	CH-GD-2017-2	China	Mar-17
MG428680	CH-GDLZ01-2017	China	16-Jun-17
MG428681	CH-GDLZ02-2017	China	17-Jun-17
MG428682	CH-GDQC-2017	China	20-Jun-17
MG428683	CH-GDYD-2017	China	30-Jun-17
MG428684	CH-GDYS01-2017	China	15-Jul-17
MG428685	CH-GDYS02-2017	China	20-Jul-17
**MH316113**	**GD04/2017**	**China**	**Sep-17**
**MH316114**	**GD01/2017**	**China**	**Sep-17**
**MH316115**	**GD03/2017**	**China**	**Sep-17**
**MH316116**	**GD05/2017**	**China**	**Sep-17**
**MH316117**	**GD06/2017**	**China**	**Sep-17**
MF460448	AH01-CH-2016	China	Nov-16
MF460449	AH02-CH-2017	China	Jun-17
MF967574	HeB01-2017	China	Aug-17
MF615501	011_15	Brazil	Jan-15
MF615503	075_16	Brazil	May-16
MF615504	077_16	Brazil	May-16
MF615505	078_16	Brazil	May-16
MF615506	128_15	Brazil	Aug-15
MF615507	235_15	Brazil	Oct-15

Locus	Strain	Country	Collection date
MF615508	244_15	Brazil	Nov-15
MF615509	245_15	Brazil	Nov-15
MF615510	282_15	Brazil	Nov-15
MH704432	SVA/VIT/3187/2018	Viet Nam	2018
MG765550	SVA/CHN/01/2017	China	27-Jun-17
MG765551	SVA/CHN/02/2017	China	25-Jul-17
MG765552	SVA/CHN/03/2017	China	7-Jul-17
MG765553	SVA/CHN/04/2017	China	25-Jun-17
MG765554	SVA/CHN/05/2017	China	2-Sep-17
MG765555	SVA/CHN/06/2017	China	1-Sep-17
MG765556	SVA/CHN/07/2017	China	19-Jul-17
MG765557	SVA/CHN/08/2017	China	15-Jun-17
MG765558	SVA/CHN/09/2017	China	29-Aug-17
MG765559	SVA/CHN/10/2017	China	5-May-17
MG765560	SVA/CHN/11/2017	China	3-May-17
MG765561	SVA/CHN/12/2017	China	26-Apr-17
MG765562	SVA/CHN/13/2017	China	27-Apr-17
MG765563	SVA/CHN/14/2017	China	26-Apr-17
MG765564	SVA/CHN/15/2017	China	19-Apr-17
MG765565	SVA/CHN/16/2017	China	7-Jul-17
MG765566	SVA/CHN/17/2017	China	16-Feb-17
MG983756	CHhb17	China	17
MH490944	SVA CH/FuJ/2017	China	Jul-17
MK256736	Porcine/GXT/92/2018	China	3-Jun-18
MH064433	HN01-2017	China	Nov-17
MH064434	HN02-2017	China	Nov-17
MH064435	LNSY01-2017	China	Dec-17
MH064436	HBWH-JX2017	China	Dec-17
MK170054	GXHZH-1	China	2018
MK170055	GXHZH-91.3	China	2018
MK170056	HN-HB20	China	2017
MH844686	1/2018/HZ/China	China	2018
MH844688	1/2018/HB/China	China	2018
**MK463618**	**GD-S5/2018**	**China**	**Mar-18**
**MK463619**	**GD-S4/2018**	**China**	**Mar-18**
MH588717	Sichuan HS-01	China	20-May-18
MH716015	SVV-SC-01	China	Jan-18
MH779611	SVV-CH-SD	China	1-Mar-18
MH817445	HS-02	China	20-May-18
MH817446	HS-03	China	20-May-18
MK252002	SVA/GD2018	China	2018
MK039162	SVA/GX/CH/2018	China	Sep-18
MK284514	CH-GDFS-2018	China	9-Nov-18

Locus	Strain	Country	Collection date
MK284515	CH-GDJY-2018	China	16-May-18
MH634506	SVA/US/CA/17-42-3D/2017	USA	2017
MH634507	SVA/US/CA/17-65-4D/2017	USA	2017
MH634508	SVA/US/CA/17-59-3D/2017	USA	2017
MH634509	SVA/US/CA/17-60-2D/2017	USA	2017
MH634510	SVA/US/CA/17-06-3D/2017	USA	2017
MH634511	SVA/US/CA/17-57-11D/2017	USA	2017
MH634512	SVA/US/CA/17-38-5D/2017	USA	2017
MH634513	SVA/US/CA/17-95-9D/2017	USA	2017
MH634514	SVA/US/CA/17-28-4D/2017	USA	2017
MH634515	SVA/US/CA/17-58-2D/2017	USA	2017
MH634516	SVA/US/CA/17-97-3D/2017	USA	2017
MH634517	SVA/US/CA/17-96-4D/2017	USA	2017
MH634518	SVA/US/CA/17-43-3D/2017	USA	2017
MH634519	SVA/US/CA/17-06-5D/2017	USA	2017
MH634520	SVA/US/CA/17-43-5D/2017	USA	2017
MH634521	SVA/US/CA/17-94-9D/2017	USA	2017
MH634522	SVA/US/CA/17-96-3D/2017	USA	2017
MH634523	SVA/US/IL/01-8/2015	USA	2015
MH634524	SVA/US/IL/01-9/2015	USA	2015
MH634525	SVA/US/IL/14-7a/2015	USA	2015
MH634526	SVA/US/IL/46-1a/2015	USA	2015
MH634527	SVA/US/IL/51-4/2015	USA	2015
MH634528	SVA/US/IL/51-6/2015	USA	2015
MH634529	SVA/US/IL/51-7/2015	USA	2015
MH634530	SVA/US/IL/72-1/2015	USA	2015
MH634531	SVA/US/IL/72-2/2015	USA	2015
MH634532	SVA/US/IL/72-3/2015	USA	2015
MH634533	SVA/US/IL/81-4a/2015	USA	2015
MH634534	SVA/US/IL/81-5a/2015	USA	2015
MK357115	HeNZMD-1/2018	China	13-Nov-18
MK357116	HeNNY-1/2018	China	24-Nov-18
MK357117	HeNKF-1/2018	China	26-Nov-18
**MK802890**	**GD-S2/2018**	**China**	**Mar-18**
**MK802891**	**GD-S3/2018**	**China**	**Mar-18**
**MK802892**	**GD-S1/2018**	**China**	**Mar-18**
MN233017	USA/IL00-66289/2000	USA	25-Aug-00
MN233018	USA/CA01-131395/2001	USA	3-Oct-01
MN233019	USA/IL01-84124/2001	USA	2001
MN233020	USA/SC05-363649/2005	USA	3-Mar-05
MN233021	USA/IA05-401302/2005	USA	4-Oct-05
MN233022	USA/TN06-429971/2006	USA	29-Mar-06
MN233023	CAN/07-503297/2007	Canada	28-Jun-07

Locus	Strain	Country	Collection date
MN233024	USA/MO15-029085/2015	USA	15-Sep-15
MN233025	USA/KS15-031348/2015	USA	2-Oct-15
MN233026	USA/NC88-23626/1988	USA	Mar-88
MN233027	USA/MN88-36695/1988	USA	5-Jul-88
MN233028	USA/89-47552/1989	USA	28-Aug-89
MN233029	USA/NJ90-10324/1989	USA	14-Dec-89
MN233030	USA/IA90-23664/1990	USA	1990
MN233031	USA/IL92-48963/1992	USA	16-Sep-92
MN233032	USA/IL94-9356/1993	USA	16-Dec-93
MN233033	USA/MN99-29256/1999	USA	16-Jul-99
MN233034	USA/LA97-98061/1997	USA	25-Nov-97
MK333629	SVA/USA/MN/004/2015	USA	31-Aug-15
MK333630	SVA/USA/MN/005/2015	USA	4-Sep-15
MK333631	SVA/USA/MN/006/2015	USA	4-Sep-15
MK333632	SVA/USA/MN/007/2015	USA	30-Sep-15
MK333633	SVA/USA/MN/009/2016	USA	13-Jul-16
MK333634	SVA/USA/MN/010/2016	USA	14-Jul-16
MK333635	SVA/USA/MN/011/2016	USA	14-Jul-16
MK333636	SVA/USA/MN/012/2016	USA	15-Jul-16
MK333637	SVA/USA/MN/013/2016	USA	2-Aug-16
MN433300	SDta/2018	China	Jan-18
MN164664	MN_US_2015	USA	2015
MN812938	USA/IA89-47552/1989	USA	28-Aug-89
MN812943	USA/TN06-00310/2006	USA	14-Mar-06
MN812944	USA/MN07-00487/2007	USA	26-Jun-07
MN812945	USA/MN07-00488/2007	USA	26-Jun-07
MN812946	USA/IA09-34037/2009	USA	14-Dec-09
MN812947	USA/HI13-007758/2013	USA	28-Feb-13
MN812948	USA/HI13-019514/2013	USA	28-May-13
MN812949	USA/HI15-021004/2015	USA	29-Jun-15
MN812950	USA/GA15-022479/2015	USA	14-Jul-15
MN812951	USA/ND15-029655/2015	USA	17-Sep-15
MN812952	USA/GA15-187/2015	USA	3-Jul-15
MN812953	USA/MI15-21/2015	USA	28-Sep-15
MN812954	USA/IL15-229/2015	USA	9-Dec-15
MN812955	USA/CA15-52/2015	USA	16-Oct-15
MN812956	USA/IA15-64/2015	USA	11-Sep-15
MN812957	USA/MI15-7/2015	USA	28-Oct-15
MN812958	USA/MI16-038766/2016	USA	22-Nov-16
MN812959	USA/MI17-011956/2017	USA	10-Apr-17
MN812960	USA/WI17-014775/2017	USA	8-May-17
MN017170	GDHY/2018	China	15-Sep-18
MN423333	CH-GDZQ-2018	China	2018

Locus	Strain	Country	Collection date
MN423334	CH-GDZQ-2018-1	China	2018
MN887248	CH-GDZS-2019	China	May-19
MN887249	CH-GDMZ-2019	China	Nov-19
MN887250	CH-GDHZ01-2019	China	Aug-19
MN887251	CH-GDHZ02-2019	China	Dec-19
MN615881	SVA/GD/China/2018	China	Oct-18
MN882351	CH-JX-JJ06	China	17-Feb-18
MN882352	CH-ZJ-JH05	China	26-Dec-18
MN882353	CH-JX-NC104	China	19-Sep-18
MN882354	CH-ZJ-LS203	China	15-Oct-18
MN882355	CH-HuN-YZH332	China	19-Mar-19
MN882356	CH-GD-FS121	China	27-Feb-19
MN882357	CH-LN-FX2212	China	26-Jan-18
MN882358	CH-HB-BD1011	China	13-May-18
MN882359	CH-HB-XT10	China	17-Aug-18
MN882360	CH-SD-WF9	China	14-Sep-18
MN882361	CH-JS-YC5478	China	28-Mar-19
MN882362	CH-HuB-XG007	China	21-May-19
MN700930	SVV-SC-MS	China	Jul-18
MN781981	CH-GDZQ-1	China	2018
MN781982	CH-GDSG-2018-1	China	2018
MN781983	CH-GDSG-2018-2	China	2018
MN781984	CH-GDSG-2018-3	China	2018
MN922286	CH-HuB-2017	China	11-Sep-17
MN885796	HeNXX/swine/2017	China	2017
MT457474	CH-GX-01-2019	China	2019
MT360257	SVA/CA/CAN2011/2011	Canada	2011
MT360258	SVA/US/SVV-001-P3/2002	USA	2002
MT360259	SVA/US-HI/NADC40/2012	USA	2012
MT360260	SVA/US-IA/IA2015/2015	USA	2015
MT360261	SVA/US-NC/NC2015/2015	USA	2015
MT360262	SVA/US-SD/SD2015/2015	USA	2015
**MW117126**	**GX02/2020**	**China**	**Mar-20**
**MW117127**	**GX04/2020**	**China**	**Mar-20**
**MW117128**	**GX84/2020**	**China**	**Mar-20**
**MW117129**	**GX94/2020**	**China**	**Mar-20**

Table S2. The detailed information of SVA recombinants.

Recombinant sequence	Minor parental sequence	Major parental sequence	RDP	GENECONV	Bootscan	Maxchi	Chimaera	SiSscan	3Seq
MH316113	KX377924	MG765556	1.33997E-08	4.12643E-05	No significant	2.29944E-09	No significant	1.08224E-17	4.72E-30
MH316113	KX751945	MG765556	No significant	2.14112E-07	No significant	4.84529E-09	3.13807E-09	1.67286E-08	4.55E-17
MK357117	MK357115	MN781982	5.11815E-18	1.59499E-15	5.64695E-16	1.25903E-09	7.18976E-10	1.40266E-13	3.16E-22
MN887249	MK802891	MW117127	3.72849E-11	1.01212E-10	No significant	8.00118E-08	2.17375E-08	5.37091E-09	5.84E-15
MK357116	MK357115	MH064434	1.20879E-13	1.10099E-10	9.63409E-13	4.19969E-08	5.04064E-07	1.1982E-10	1.39E-11
MG765559	MH316114	MG765560	1.53071E-07	4.919E-08	6.25861E-06	1.25491E-07	1.93904E-07	7.95836E-07	2.42E-11

Table S3. Details and results of AMOVA for assessing the genetic differentiation among and within clades I and II.

Source of variation	d.f.	Sum of squares	Variance components	Percentage of variation
Among clades	1	3654.585	102.41391****	56.08
Within clades	231	18528.05	80.20801	43.92
Total	232	22182.635	182.62192	

d.f., Degrees of freedom; ****, significant at $P < 0.0001$.

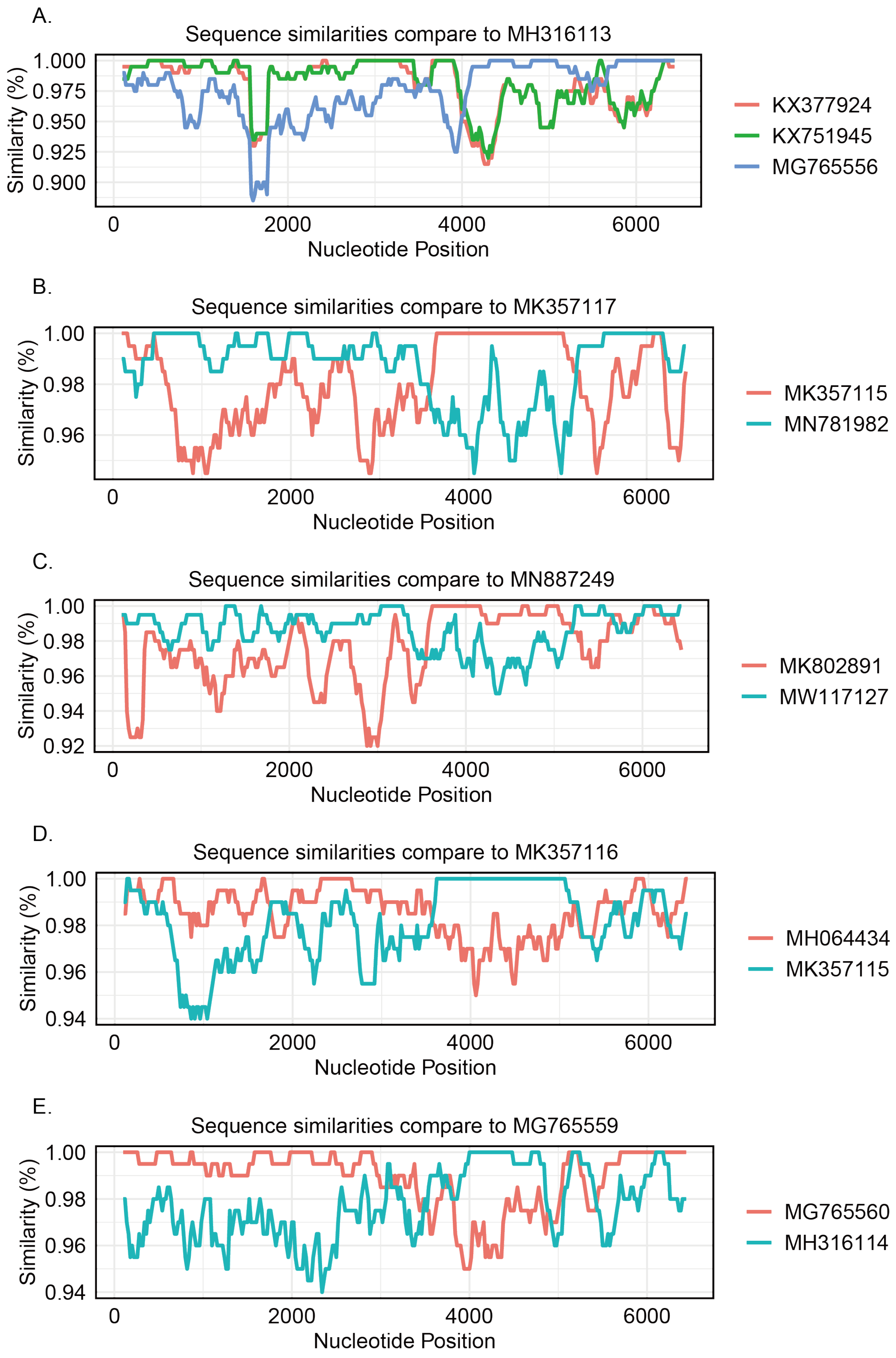

Fig. S1. Recombination analysis. Each curve is a comparison between recombinant and its parental strain. The *x*-axis represents the nucleotide

positions of SVA genome. The *y*-axis refers to percentage identity with a sliding window size of 200 bp and with a step size of 20 bp between plots. A. Recombination analysis of GD04/2017 strain. Similarity plots of HB-CH-2016 (KX377924, parental strain), CH-LX-01-2016 (KX751945, parental strain), and SVA/CHN/07/2017 (MG765556, parental strain) against GD04/2017 (MH316113, recombinant). B. Recombination analysis of HeNKF-1/2018 strain. Similarity plots of HeNZMD-1/2018 (MK357115, parental strain) and CH-GDSG-2018-1 (MN781982, parental strain) against HeNKF-1/2018 (MK357117, recombinant). C. Recombination analysis of CH-GDMZ-2019 strain. Similarity plots of GD-S3/2018 (MK802891, parental strain) and GX04/2020 (MW117127, parental strain) against CH-GDMZ-2019 (MN887249, recombinant). D. Recombination analysis of HeNNY-1/2018 strain. Similarity plots of HeNZMD-1/2018 (MK357115, parental strain) and HN02-2017 (MH064434, parental strain) against HeNNY-1/2018 (MK357116, recombinant). E. Recombination analysis of SVA/CHN/10/2017 strain. Similarity plots of GD01/2017 (MH316114, parental strain) and SVA/CHN/11/2017 (MG765560, parental strain) against SVA/CHN/10/2017 (MG765559, recombinant).

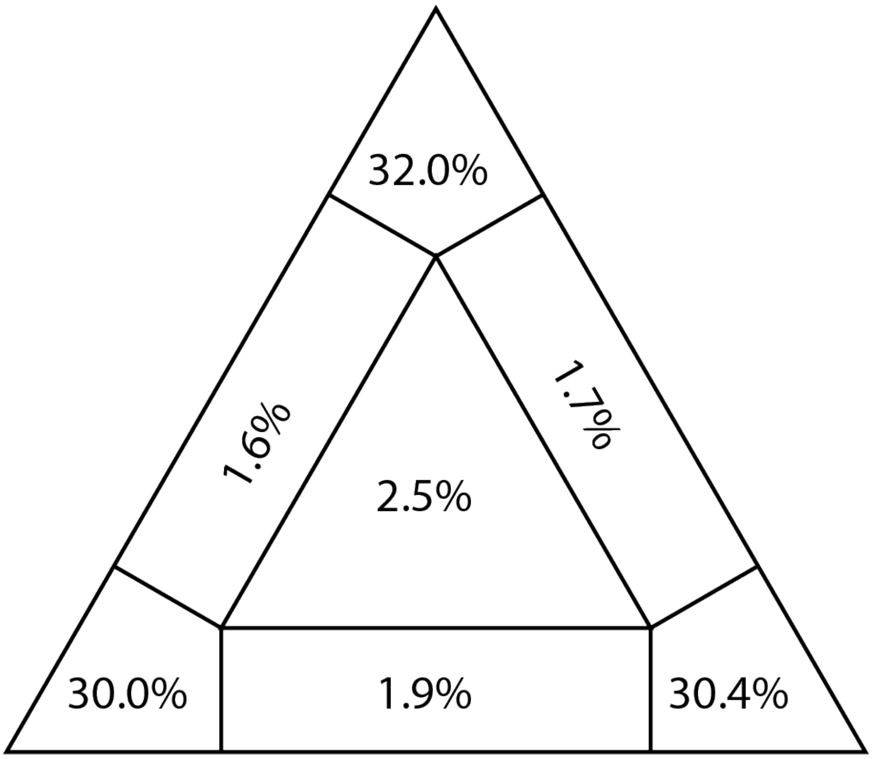

Fig. S2. The phylogenetic signal detection of SVA dataset using likelihood-mapping analysis. The triangle is the two-dimensional simplex graph supporting different evolutionary information: alternative topologies (tips), unresolved quartets (center) and partly resolved quartets (edges).

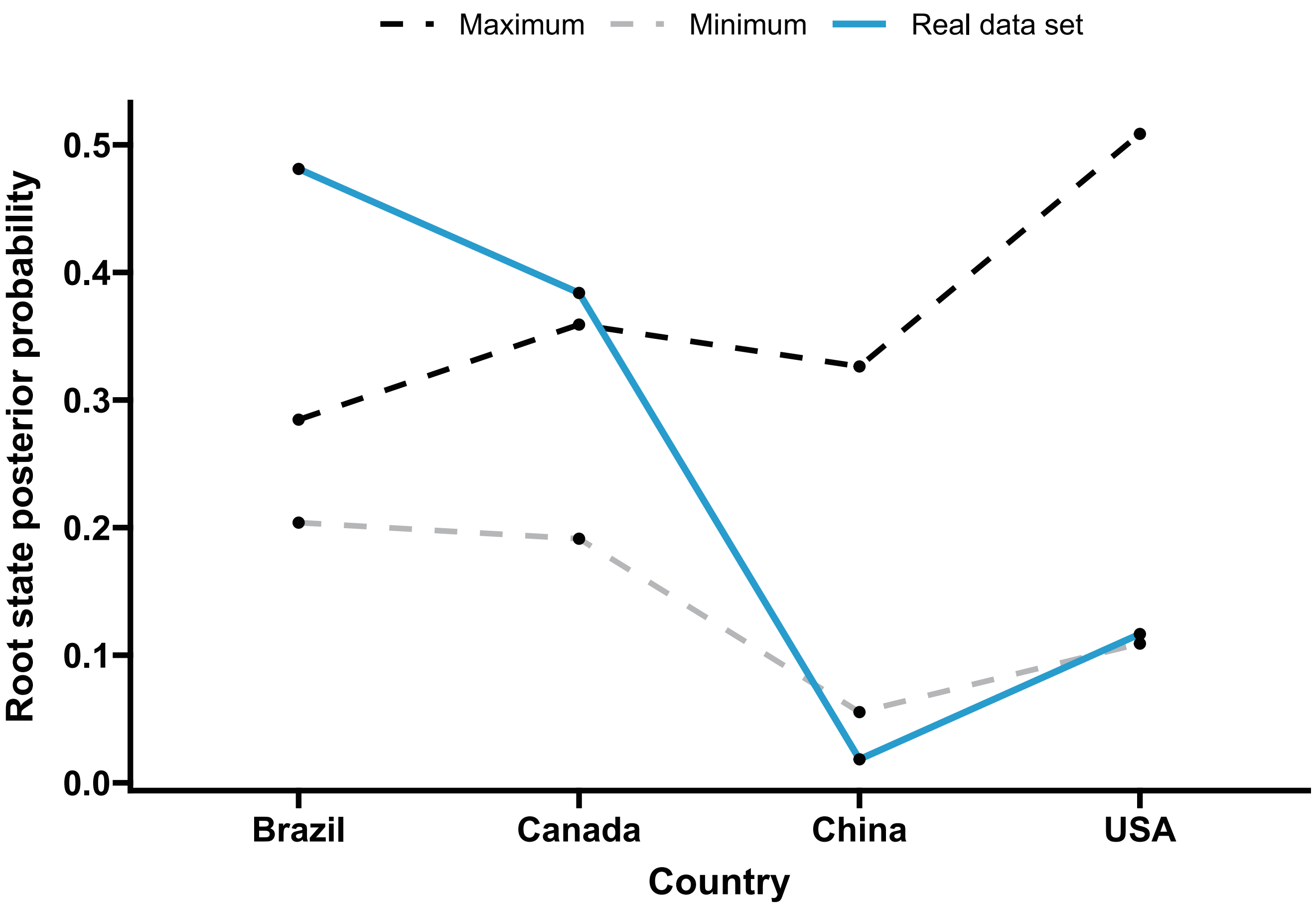

Fig. S3. Results of the location-randomization analysis. The y-axis represents the root state posterior probability (RSPP). The maximum and minimum values for RSPPs estimated from 20 location-randomized subsamples datasets are indicated by the black and gray dashed lines, respectively. The values for RSPPs estimated from the original data set are shown by the solid blue line.
